# Cholera

**Published:** 1849-07

**Authors:** 


### CHOLERA.

We are indebted to the Cincinnati Gazette for a tabular statement of the rise and progress of the epidemic in this city. The interments for the last four or five days have been added to the table, but not to the general aggregate; yet the number additional is so small, that the general result as presented, is changed but little.

By the last day's report, August 6th, the number of interments from Cholera is only 6. This, we think, will justify us in saying, that as an epidemic, the disease no longer exists in our city. Indeed, for the last five days, (this being the 10th,) the average is not over the above. We presume that in no place where it has prevailed so extensively, so few left to avoid the disease, and in no place was the regular business less interrupted. It came among us at a time when business is generally somewhat dull; yet we can say, that for ourself we have not been more constantly occupied, professionally, for the last three years, than during the same months. We are glad however, to find that every department of business begins to wear a more cheerful aspect. The fatality has truly been heart-rending, and among the foreign part of our population, has it been most emphatically so. We are satisfied, by a careful observation, during the entire progress of the disease, that a large majority of those attacked, and who were treated promptly and energetically, got well. This disease, when taken early, was generally easily managed, it being almost always preceded by some disturbance of the stomach or bowels, that was admonitory of its approach.

Of the many theories advanced to account for its approach and prevalence, we have but little to say, having never seen any but failed, in our opinion, to account for the different laws which appear at different times and places, to govern this disease. We therefore would merely present our readers with the rise and progress of the disease in our city, and give, in addition, a meteorological table, showing the condition of the weather during the entire period, and for nine days preceding the outbreak of the disease as an epidemic. We put the two tables together, that physicians and others may make such deductions therefrom, as the facts may warrant.—[Cin. Ed.

